# Indoxacarb poisoning: A rare presentation as methemoglobinaemia

**DOI:** 10.4103/0019-5049.65373

**Published:** 2010

**Authors:** Roopam Chhabra, Ishwar Singh, Mansi Tandon, Ram Babu

**Affiliations:** Department of Anaesthesiology and Intensive Care, Jaipur Golden Hospital, New Delhi, India; 1 Department of Medicine, Jaipur Golden Hospital, New Delhi, India

**Keywords:** Indoxacarb, methemoglobinaemia, methylene blue

## Abstract

Indoxacarb is a broad-spectrum non-organophosphorus oxidiazine insecticide widely used in farming. Once absorbed it acts on sodium channels and blocks the flow of sodium ions. We report a case of indoxacarb poisoning in a farmer following suicidal consumption, manifested as unconsciousness, cyanosis and stationary SpO_2_ values. Methemoglobinaemia was suspected on clinical presentation which was successfully managed with inj. methylene blue and other symptomatic and supportive treatment.

## INTRODUCTION

Indoxacarb is an oxidiazine[1] compound and a sodium channel blocker. It is a broad-spectrum insecticide[1] widely used in commercial and farm planting for the control of certain insects, i.e. moth, leaf hopper and fruit worm.

## CASE REPORT

A 52-year-old farmer came to our intensive care unit with an alleged history of consumption of an unknown pesticide (suicidal attempt) while working in the field during the day hours. As per records he was given gastric lavage and inj. atropine in a local hospital and then referred to our hospital. On admission he was cyanosed and unresponsive with a Glasgow coma scale (GCS) of 8/15. He was immediately intubated and put on controlled mechanical ventilatory support with a FiO_2_ of 1.0. The noninvasive blood pressure (NIBP) was 80/60 mmHg, pulse rate (PR) was 130 per min and SpO_2_ was 83%. The pupils were bilaterally dilated and were sluggishly reacting to light. The right internal jugular vein was cannulated and central venous pressure (CVP) was 3 cm of water. Intravenous fluids were given to keep a CVP of 10 cm of water or a urine output of 50 ml/h. Considering the possibility of organophosphorus poisoning, inj. atropine[2] 2 mg i.v. and inj. pralidoxime[2] 2 g i.v. (dose 25–50 mg/kg) were given over a period of 15 min. The investigations like complete blood count, kidney function test, liver function test, serum electrolytes and serum cholinesterase were sent. While sampling, the arterial blood was found to be chocolate brown coloured,[3] which did not change its colour even on deliberate exposure to room air. Another arterial sample was analysed and revealed pH 7.25, PaCO_2_ 38, PaO_2_ 245, HCO_3_ 16.7 and SaO_2_ 88%. It was observed that despite continuing mechanical ventilation with 100% O_2_, the SpO_2_ value did not rise and remained stationary at 83–84% for another hour.

The patient’s relatives were thoroughly questioned again about the poison and later on they could bring the bottle of poison consumed. It was indoxacarb (14.5% SC *Avaunt*), which is a non-organophosphorus oxidiazine insecticide.[1,4] Therefore inj. pralidoxime and inj. atropine were not repeated. Considering the cyanosis, stationary SpO_2_, chocolate brown colour of arterial blood and disproportionately high PaO_2_ on arterial blood gas (ABG) analysis, the possibility of methemoglobinaemia[5] was suspected. As the facility for determining methemoglobin levels was unavailable, we started the treatment on the basis of clinical suspicion. Injection methylene blue[3,5] 150 mg diluted in 100 ml saline (dose 2 mg/kg, preparation 1%, Samarth Life Sciences pvt. Ltd., Solan Himachal Pradesh 174101) was given over a period of 10 min intravenously. Inj. ascorbic acid[3,6] (vitamin C) 500 mg in 5% dextrose was also started at a rate of 100 ml/h. Within an hour of giving inj. methylene blue, the patient started improving in terms of better sensorium and an increased SpO_2_ value (90%). Repeat ABG analysis showed pH 7.47, PaCO_2_ 30, PaO_2_ 315, HCO_3_ 21.8 and SaO_2_ 98% at a FiO_2_ of 1.0. Thereafter, FiO_2_ was reduced to 0.6 and inj. methylene blue 70 mg (dose 1 mg/kg) and ascorbic acid 500 mg in 5% dextrose were continued at 12-h intervals. The next day SpO_2_ value rose to 92%; ABG analysis revealed pH 7.48, PaCO_2_ 32, PaO_2_ 340, HCO_3_ 22.3 and SaO_2_ 100% at a FiO_2_ of 0.6 and he became responsive to verbal commands. He was put on the synchronised intermittent mandatory ventilation (SIMV) mode with a FiO_2_ of 0.5 and a pressure support of 12 cm of H_2_O. On day 3, the SpO_2_ value rose to 95% and he became fully conscious. He was given a T-piece trial with O_2_ at a rate of 5 l/min. ABG showed a pH 7.46, PaCO_2_ 36, PaO_2_ 410, HCO_3_ 21.6 and SaO_2_ 100%. The next day he was extubated and was kept on face mask with O_2_ at a rate of 5 l/min. On day 5 with stable haemodynamics and GCS 15/15 he was shifted to ward.

## DISCUSSION

Indoxacarb insecticide is formulated as 30% active water-soluble granule. The oral LD_50_[1] is 1800 mg/kg and dermal LD_50_[1] is >5000 mg/kg. It affects insects by either direct exposure of spray droplets or through ingestion of treated foliage and fruit. Once absorbed, it kills the organism by blocking the flow of sodium ions.[1,4] This results in impaired nerve function, cessation of feeding, paralysis and death in insects.

Methemoglobin[7] (MetHb) is generated by the oxidation of haem iron moieties to the ferric state causing characteristic bluish brown colour resembling cyanosis. MetHb has such a high affinity to O_2_ that virtually no O_2_ is delivered to tissues and the O_2_ dissociation curve is shifted towards left. Methemoglobinaemia[5] should be suspected in patients with hypoxic symptoms who appear cyanotic, though PaO_2_ levels on ABG analysis are sufficiently high to fully saturate the haemoglobin. The characteristic chocolate brown appearance of freshly drawn blood can be a critical clue.[3] The SpO_2_ value around 85% is because of the typical light absorbance spectra[8] of MetHb. Normally MetHb levels[3,5–7] are less than 1%. The cyanosis[3,5–7] usually manifests at a level of 15% and treatment is warranted[3,5–7] at levels above 30% while levels >60% are considered to be lethal.[3,5–7]

Methemoglobinaemia is treated with methylene blue, 1–2 mg/kg, administered slowly. If cyanosis persists, the dose may be repeated at an hourly interval to a maximum[5] of 7 mg/kg/day. The maintenance dose[5] of methylene blue is 1 mg/kg twice or three times a day. Other supportive measures include the administration of vitamin C and correction of the metabolic abnormalities.

In our patient, methemoglobinaemia had occurred following the ingestion of indoxacarb insecticide which was identified and treated on the basis of clinical suspicion and ABG analysis. Further literature research revealed a similar case report[9] published in year 2008 in IJCCM.

Therefore, it can be substantiated that indoxacarb, an oxidiazine insecticide, also manifests its toxicity in humans in the form of methemoglobinaemia. As in our patient, it could be successfully managed with intravenous methylene blue and other supportive and symptomatic treatment. As indoxacarb is a commonly used insecticide, the clinicians working in ICUs should be able to recognise and manage it.
